# Resuscitation of viable but non‐culturable bacteria to enhance the cellulose‐degrading capability of bacterial community in composting

**DOI:** 10.1111/1751-7915.13256

**Published:** 2018-03-14

**Authors:** Xiaomei Su, Shuo Zhang, Rongwu Mei, Yu Zhang, Muhammad Zaffar Hashmi, Jingjing Liu, Hongjun Lin, Linxian Ding, Faqian Sun

**Affiliations:** ^1^ College of Geography and Environmental Science Zhejiang Normal University Jinhua 321004 China; ^2^ Environmental Science Research and Design Institute of Zhejiang Province Hangzhou 310007 China; ^3^ Department of Meteorology COMSATS Institute of Information Technology Islamabad 44000 Pakistan; ^4^ Department of Architecture and Resources Engineering Jiangxi University of Science and Technology Nanchang 310013 China

## Abstract

Nowadays, much of what we know regarding the isolated cellulolytic bacteria comes from the conventional plate separation techniques. However, the culturability of many bacterial species is controlled by resuscitation‐promoting factors (Rpfs) due to entering a viable but non‐culturable (VBNC) state. Therefore, in this study, Rpf from *Micrococcus luteus* was added in the culture medium to evaluate its role in bacterial isolation and enhanced effects on cellulose‐degrading capability of bacterial community in the compost. It was found that *Proteobacteria* and *Actinobacteria* were two main phyla in the compost sample. The introduction of Rpf could isolate some unique bacterial species. The cellulase activity of enrichment cultures with and without Rpf treatment revealed that Rpf treatment significantly enhanced cellulase activity. Ten isolates unique in Rpf addition displayed carboxymethyl‐cellulase (CMCase) activity, while six isolates possessed filter paper cellulase (FPCase) activity. This study provides new insights into broader cellulose degraders, which could be utilized for enhancing cellulosic waste treatment.

## Introduction

Cellulosic biomass is the most abundant raw material with great potential for producing many commodity chemicals, as well as bioenergy (Rastogi *et al*., 2010). Currently, thermophilic bioprocessing of cellulosic biomass to bioproducts and biofuels is a promising way for agricultural and municipal cellulosic waste (Sun *et al*., 2016). The insoluble cellulose components can be converted to soluble sugar by cellulolytic microorganisms (Manfredi *et al*., 2015). Many bacteria belonging to several different phyla, such as *Proteobacteria*,* Actinobacteria*,* Firmicutes*,* Bacteroidetes* and *Chloroflexi,* have been reported with cellulosic activities (Medie *et al*., 2012; Koeck *et al*., 2014). Moreover, Medie *et al*. (2012) reported that ~40% of the genomes of sequenced bacteria encoded at least one cellulase gene by analysis of ~1500 complete bacterial genomes. A huge amount of research has gone into elucidating the cellulose‐degrading bacterial communities by various methods including cultivation and different molecular techniques (Field *et al*., 2010; Rastogi *et al*., 2010; Amore *et al*., 2012; Gupta *et al*., 2012; Liu *et al*., 2014; Manfredi *et al*., 2015). To elucidate the function and genotype of microorganisms, a great need has existed for pure cultures isolation. In general, much of what we know regarding the pure cellulolytic microorganisms comes from the conventional plate separation techniques.

Definitely, our understanding of the degradation of cellulosic material is still limited, because most of the microbial diversity cannot be cultivated using conventional bacteriological media and remains inaccessible (Epstein, 2013). As well known, numerous bacteria including pollutant‐degrading bacteria can enter into a viable but non‐culturable (VBNC) state when exposed to adverse environmental conditions (Colwell *et al*., 1985; Oliver, 2010; Su *et al*., 2013a,b; Su *et al*., 2015a,b,c,d). For example, it was previously reported that a novel biphenyl‐degrading bacterium *Rhodococcus biphenylivorans* could enter the VBNC state under oligotrophic and low‐temperature conditions (Su *et al*., 2015a,b,c,d). Most investigators believe the VBNC state is a survival strategy which contributes to long‐term maintenance of cells viability (Keep *et al*., 2006; Oliver, 2010). Clearly, understanding and controlling the entry and exit from the VBNC state are crucial for functional microorganisms in cellulosic biomass. Therefore, more investigations are required for resuscitating potential cellulose‐degrading bacteria in the VBNC state.

A breakthrough development of recovering VBNC bacteria is the discovery of resuscitation‐promoting factors (Rpfs; Mukamolova *et al*., 1998, 2002; Lennon and Jones, 2011). In particular, over the past decade, researchers have achieved major advances in the bacterial activation by Rpf from *Micrococcus luteus* which is the founder member of Rpfs (Cohen Gonsaud *et al*., 2005; Keep *et al*., 2006; Mukamolova *et al*., 2006). This Rpf is a 16–19 kDa protein with muralytic activity which can facilitate cell division and regrowth at very low picomolar concentrations by remodelling the cell envelope of VBNC cells (Mukamolova *et al*., 1998; Lennon and Jones, 2011). Especially, the resuscitating function of the Rpf in the *Actinobacteria* has been verified in a wide variety of bacterial species, and Rpf homologues are found in several high GC Gram‐positive bacteria belonging to the *Actinobacteria* (Keep *et al*., 2006; Mukamolova *et al*., 2006). Overall, the culturability of many members of phylum *Actinobacteria* is controlled by Rpfs.

Notably, *Actinobacteria* comprise many well‐known cellulose degraders, including *Streptomyces*,* Nocardiopsis*,* Mycobacterium*,* Arthrobacter* and *Rhodococcus* (Amore *et al*., 2012; Koeck *et al*., 2014; Deng *et al*., 2016). Moreover, cellulose is very recalcitrant and not easily accessible for the attacking microorganisms due to its insolubility and the presence of crystalline regions (Koeck *et al*., 2014). Thus, VBNC or uncultured bacteria in cellulosic biomass must be able to respond to Rpf, otherwise they will miss chances for growth and division. However, until recently, little is known about the potential cellulase activities of uncultured bacteria, as previous studies have focused on resuscitation of VBNC bacteria from the view of medicine and epidemiology (Mukamolova *et al*., 2002; Oliver, 2010; Rosser *et al*., 2017). Research is needed to explore the uncultivated bacteria which are potential cellulose degraders using the resuscitation and stimulation function of Rpf.

In this study, the microbial consortia in a representative compost sample were characterized by Illumina high‐throughput sequencing targeting the 16S rRNA gene, and then, enrichment and isolation of cellulolytic microorganisms from the compost were performed with and without Rpf treatment respectively. The effect of Rpf on cellulose‐degrading capability of bacterial community was investigated by measuring cellulolytic activity. The overall purpose of this study was to determine the resuscitation function of Rpf on cellulolytic microorganisms using culture‐dependent and culture‐independent approaches. This study will broaden our knowledge of these uncultured bacteria in cellulosic biomass decomposition.

## Results and Discussion

### Expression of Rpf in Escherichia coli and its characterization

**Figure 1:**
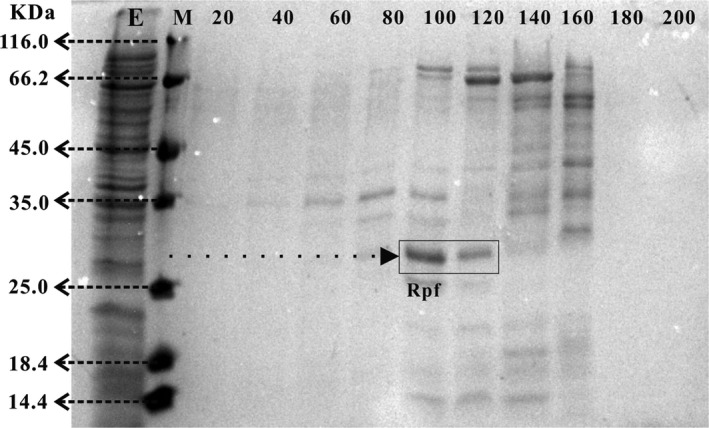
SDS‐PAGE profiles of recombinant Rpf protein after purification on a nickel‐nitrilotriacetic acid (Ni‐NTA) column with different concentrations (20–200 mM, increased in steps of 20 mM) of imidazole; Lanes: 1, E (eluent); 2, M (protein markers); 3–12, imidazole concentrations of 20–200 mM.

The histidine‐tagged recombinant Rpf protein was expressed in *E*. *coli* BL21 (DE3) and then purified on a nickel‐nitrilotriacetic acid (Ni‐NTA) column with different concentrations (20–200 mM) of imidazole. Sodium dodecyl sulphate (SDS)–polyacrylamide gel electrophoresis (PAGE) profiles of purified Rpf protein are presented in Fig. 1. The molecular mass (kDa) markers were β‐galactosidase (116.0), bovine serum albumin (66.2), ovalbumin (45.0), lactate dehydrogenase (35.0), REase Bsp981 (25.0), β‐lactoglobulin (18.4) and lysozyme (14.4). Rpf was just visible as a distinct band at imidazole concentrations of 100 and 120 mM, and the clearer band was observed at imidazole concentration of 100 mM. The clearest band was observed corresponding to a protein with an apparent molecular mass of 26–27 kDa, which was consistent with the theoretical molecular weight (26.74 kDa) of the recombinant Rpf protein. Mukamolova *et al*. (2006) reported that the molecular mass of histidine‐tagged recombinant protein was 25 kDa assessed by SDS‐PAGE. Therefore, the Rpf protein obtained at imidazole concentration of 100 mM was selected for concentration analysis and for further experiments. The concentration of recombinant Rpf protein reached 0.7218 g/l, and the peptidoglycan hydrolase activity of Rpf protein was clearly confirmed by the standard turbidimetric assay. Within 3 h after addition of Rpf, the averaged values of OD_600_ decreased from 0.86 to 0.32, whereas it decreased to 0.81 without Rpf addition. In addition, lysis zone was also observed in plate containing peptidoglycan treated with the recombinant Rpf protein (data not shown). These results indicated that the recombinant Rpf protein obtained in the study was consistent with the previous reports that the muralytic activity of Rpf is probably responsible for its resuscitation and stimulation functions (Cohen Gonsaud *et al*., 2005; Mukamolova *et al*., 2006).

### The bacterial community in the compost sample

**Figure 2:**
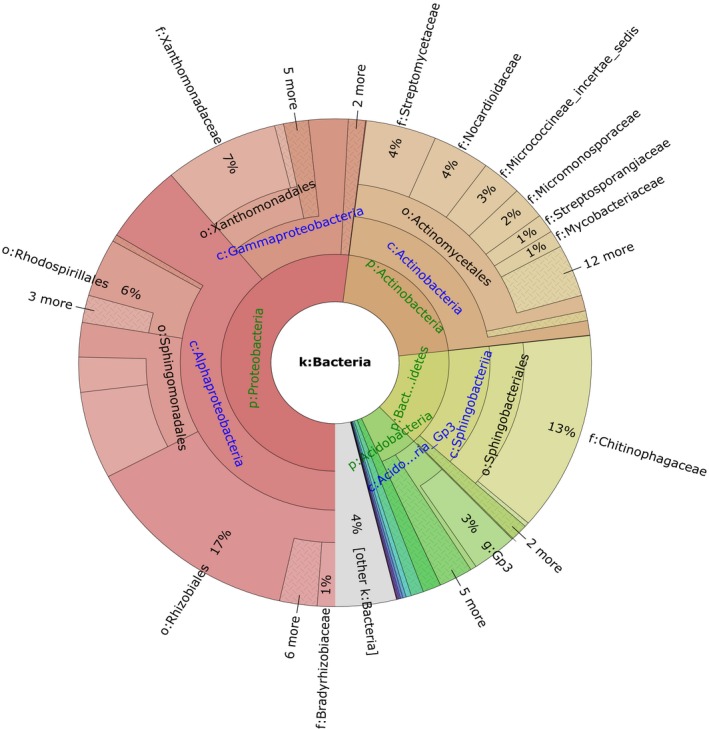
Krona chart based on the OTUs showing the relative abundance and diversity of bacterial community in the waste composting sample for the V3–V4 Illumina dataset; Circle from inside to outside stand for different classification levels.

**Figure 3:**
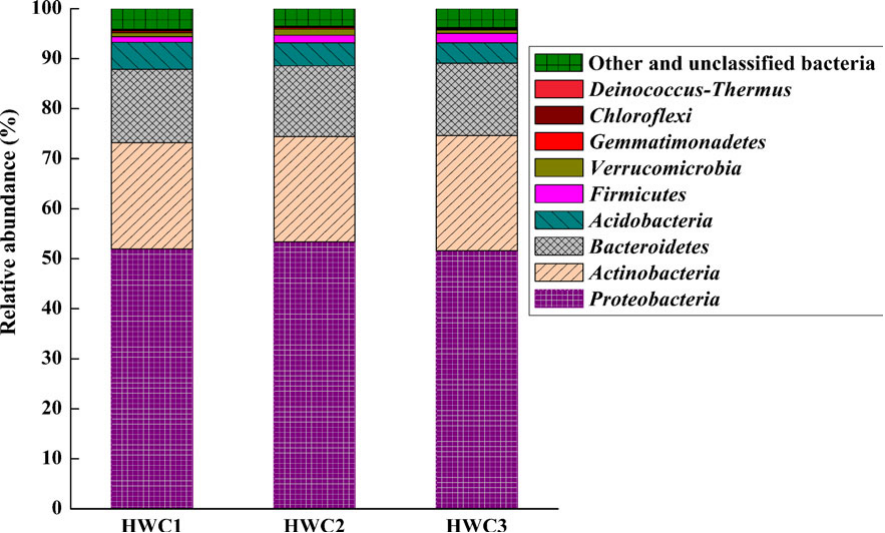
Taxonomic composition of bacterial community at the phylum level in the compost sample (> 0.1%).

**Figure 4:**
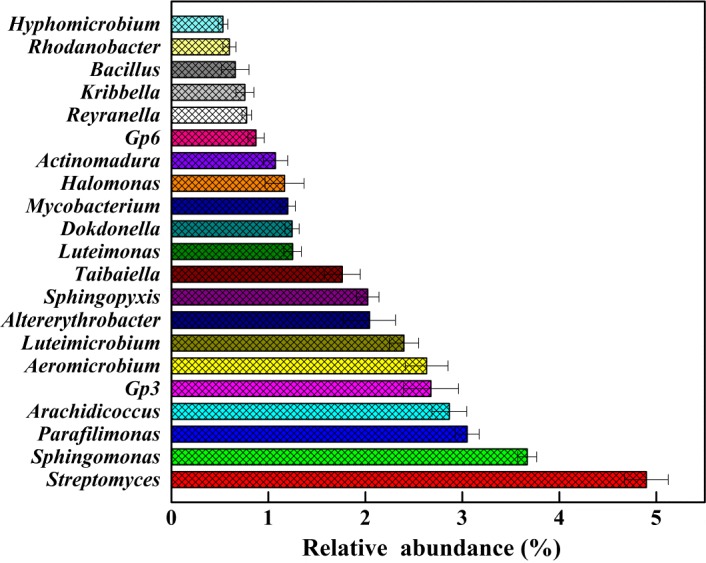
Taxonomic composition of bacterial community at the genus level in the compost sample (> 0.5%).

Illumina high‐throughput sequencing was adopted to investigate bacterial community diversity and structure of the compost samples (HWC1, HWC2 and HWC3). As listed in Table 1, the sequencing resulted in 46 642, 44 597 and 51 405 raw reads from HWC1, HWC2 and HWC3 respectively. After removing low‐quality sequences, at least 34 725 clean tags were obtained for each sample with an average length of 412 bp. The values of both clean Q20 and Q30 reads were higher than 96%, demonstrating that we obtained sequencing data of high quality. The taxon tags from HWC1, HWC2 and HWC3 were 36 439, 32 464 and 37 380 respectively. The calculated rarefaction curve approached saturation, indicating that the diversity of compost samples in sequencing library was almost covered. Bacterial species richness and diversity analysis showed that there is no significant difference in the three biological replicates. Based on the average distribution of operational taxonomic units (OTUs), the taxonomic composition of the bacterial community was demonstrated in Krona chart (Fig. 2), where circles from inside to outside represented different taxonomic levels. These bacteria can be assigned to 18 different phyla, 31 classes, 40 orders, 76 families and 110 genera. The distributions of sequences (relative abundance > 0.1%) at the phyla level are shown in Fig. 3. In total, nine identified phyla were observed, in which *Proteobacteria*,* Actinobacteria*,* Bacteroidetes* and *Acidobacteria* were the dominant phyla. Previous studies have demonstrated that *Proteobacteria* and *Actinobacteria* were key players in the compost sample, and they were also the effective phyla for organic pollutants degradation (Awasthi *et al*., 2017; Xie *et al*., 2017). Moreover, some studies also revealed similar bacterial diversity in waste composting samples by the high‐throughput sequencing (Lv *et al*., 2015; Sun *et al*., 2016). However, the abundance of *Firmicutes* obtained in our study was lower than that reported by Partanen *et al*. (2010) which indicated that *Firmicutes* was also among the most abundant phyla. At the genus level, the relative abundance greater than 0.5% was presented in Fig. 4. The most predominant genus was *Streptomyces* (4.90 ± 0.23%), followed by *Sphingomonas* (3.67 ± 0.10%), *Parafilimonas* (3.05 ± 0.12%) and *Arachidicoccus* (2.87 ± 0.18%). It is well known that *Streptomycetes* are important members of soil community and are particularly active in the biodegradation of recalcitrant organic contaminants (Inbar *et al*., 2005). Amore *et al*. (2012) found that *Streptomyces* sp. strain G12 produced the highest cellulolytic activity among different microorganisms isolated from compost. Other strains of *Streptomyces* sp. were also reported as producers of cellulolytic activity (Liu *et al*., 2014). Moreover, the genus *Sphingomonas* has been widely reported to possess diverse metabolic capabilities towards organic xenobiotics (Marchal *et al*., 2013). In addition, the genera *Parafilimonas* and *Arachidicoccus* which belonged to family *Chitinophagaceae,* a member of phylum *Bacteroidetes*, were identified recently (Kim *et al*., 2014; Madhaiyan *et al*., 2015). Therefore, these results showed that bacterial community identified in the study is quite similar to the previous publications (Marchal *et al*., 2013; Lv *et al*., 2015; Madhaiyan *et al*., 2015; Sun *et al*., 2016).

**Table 1:** Information and quality of Illumina high‐throughput sequencing data from composting samples

Samples	HWC1	HWC2	HWC3
Raw reads	46 642	44 597	51 405
Clean tags	39 193	34 725	39 847
Taxon tags	36 439	32 464	37 380
CleanQ20 (%)^a^	98.08	98.17	98.17
CleanQ30 (%)^a^	96.03	96.24	96.18
Average length (bp)	412.13	412.44	412.37
OTUs	540	528	530
Shannon	6.84	6.79	6.83
Equitability	0.754	0.751	0.755

a Clean Q20 or Q30: the percentage of bases with a phred value of > 20 or 30.

### Isolation and phylogenetic analysis of bacteria

**Figure 5:**
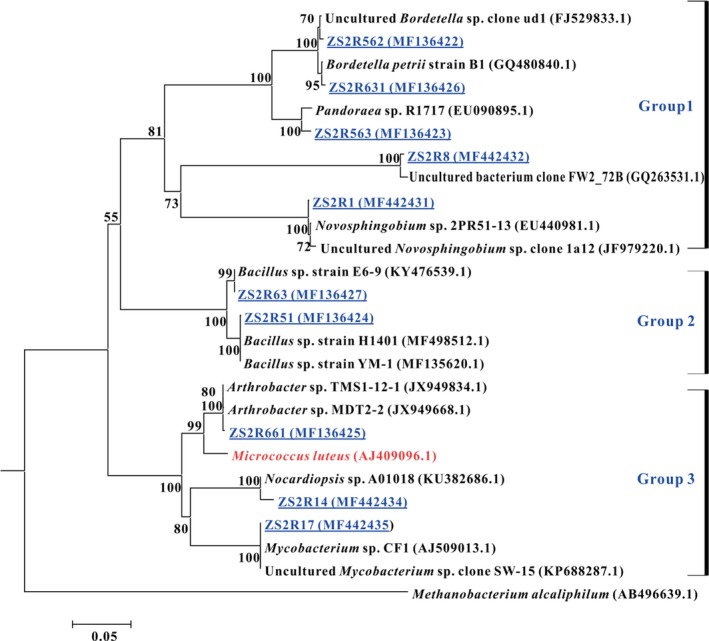
Neighbour‐joining phylogenetic tree of bacterial 16S rRNA gene sequences, including ten isolates unique to enrichment culture with Rpf addition and 15 of their most similar GenBank sequences. Bootstrap values (percentages based on 1000 replications) of above 50% are shown at the branch points. *Methanobacterium alcaliphilum* was used as an outgroup.

Using the isolation procedure described above, 21 and 11 strains were obtained in TG and CG respectively. Based on combined morphology and 16S rDNA sequence analysis, 10 of 21 strains were unique in the treatment group (TG), while no counterparts in control group (CG) were obtained. The remaining 11 strains which were existed in both TG and CG were analysed by retrieving the most closely related strains from EzbioCloud database (https://www.ezbiocloud.net/) (Table S1). Figure 5 presented an overview of 16S rRNA‐based phylogenetic tree of the isolates and the closely matching representatives obtained from GenBank. Overall, at the phylum level, the 10 isolates can be divided into three groups 1, 2 and 3 which belonged to *Proteobacteria*,* Firmicutes* and *Actinobacteria* respectively. Strains in group 1 belonged to Gram‐negative bacteria, which were well supported by the studies that Rpf also resuscitated and stimulated the growth of some Gram‐negative bacteria (Su *et al*., 2013a,b, 2015a,b,c,d). In particular, ZS2R8 was closely related to uncultured bacterium clone FW2_72B (GQ263531.1) which was detected at low‐level waste site with cellulosic contaminants (Field *et al*., 2010). Strains in group 2 were closely related to *Bacillus* sp., which were low G+C Gram‐positive bacteria. Many *Bacillus* species have been isolated earlier from compost environments and have been demonstrated to degrade cellulose, carboxymethyl‐cellulose (CMC) and filter paper (Rastogi *et al*., 2010). Strains ZS2R661, ZS2R14 and ZS2R17 in the group 3 belonging to genera *Arthrobacter*,* Nocardiopsis* and *Mycobacterium*, respectively, were closely related to *M*. *luteus*. The result is consistent to those studies reporting Rpf protein promotes the resuscitation and growth of high G+C Gram‐positive bacteria (Mukamolova *et al*., 1998; Oliver, 2010; Su *et al*., 2013a,b). Moreover, Field *et al*. (2010) observed that members of *Actinobacteria* containing known cellulose‐degrading bacteria increased in the fill‐waste interface layer, which suggested the potential capabilities of the *Actinobacteria* phylum to degrade cellulose. Additionally, our previous studies found that extracellular organic matter (EOM) from *M*. *luteus* containing the Rpf could stimulate and resuscitate uncultured bacteria belonging to phylum *Actinobacteria* (Su *et al*., 2015a,b,c,d). Therefore, the results of these cultivation studies are in line with the resuscitating and stimulating functions of Rpf on cellulose‐degrading bacteria in the compost sample.

### Effect of Rpf on extracellular cellulase activity

**Figure 6:**
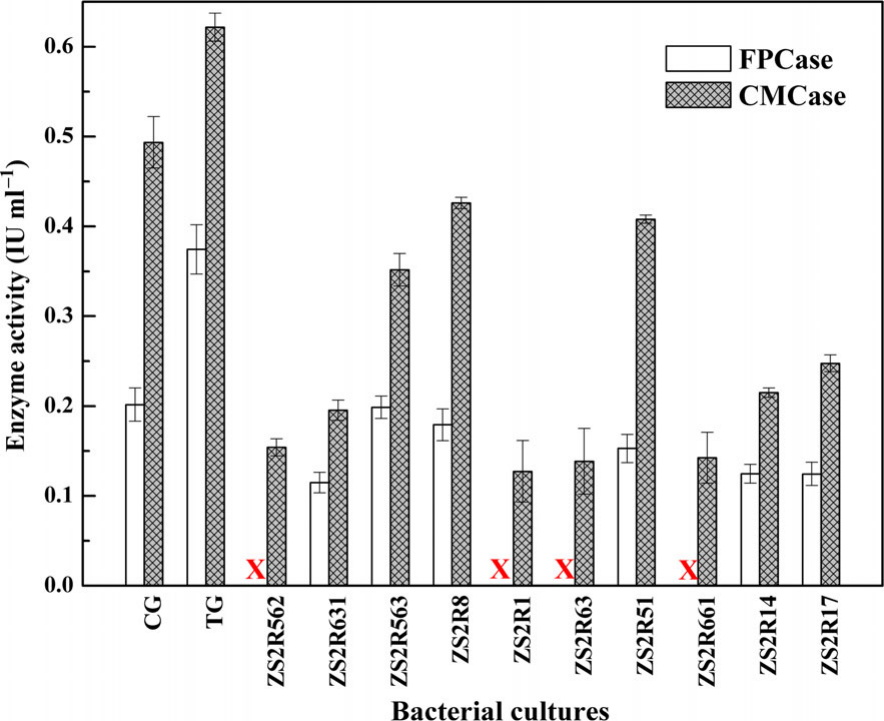
Extracellular cellulase activity of two enzymes (FPCase and CMCase) of bacterial cultures from TG and CG, as well as the ten isolates. X: no FPCase activity was detected. Each bar represents the average of three replicates in each sample with standard deviation (SD).

To gain a better understanding of the potential role of Rpf in cellulose degradation, the cellulase activity of bacterial community in TG and CG, as well as each pure isolate, was analysed and the results are presented in Fig. 6. It was indicated that filter paper cellulase (FPCase) and carboxymethyl‐cellulase (CMCase) activities in the TG were both higher than those in the CG. The activity was 0.3744 IU/ml for FPCase and 0.6217 IU/ml for CMCase in TG, while the activities were 0.2016 for FPCase and 0.4935 IU/ml for CMCase in CG. Meanwhile, the cellulase activities detected from the mixed cultures of TG and CG were higher than those produced by pure cultures. For pure isolates, all strains displayed CMCase activity ranging from 0.1274 IU/ml to 0.4261 IU/ml, while only six strains possessed FPCase activity ranging from 0.0973 IU/ml to 0.1986 IU/ml. In comparison with other strains, the two strains ZS2R63 and ZS2R51 exhibited higher CMCase activity, while the maximum FPCase activity was observed for the strain ZS2R17. Gupta *et al*. (2012) also reported similar results that enzyme assay for FPCase activity was found to be highest for CDB10, while for CMCase assay, maximum activity was found by CDB8.

Although there are many reports on cellulose degradation by pure isolates, most of them utilized optimized‐medium conditions to assay maximum cellulase activity (Amore *et al*., 2012; Manfredi *et al*., 2015; Wang *et al*., 2016). Our data on extracellular cellulase activities were generated from crude culture supernatant without optimization. Notably, most studies focused on description and characterization of the cellulose‐degrading bacteria which were obtained using the existing limited pure culture medium (Amore *et al*., 2012; Wang *et al*., 2016). For example, Manfredi *et al*. (2015) recovered 233 pure isolates from gut samples and found that only 1.7% showed both FPCase and CMCase activities. Therefore, although many microorganisms have been extensively investigated for their cellulosic activities, the well‐explored high‐efficient cellulose‐degrading bacteria are still limited due to the inability to culture a vast majority of functional microorganisms present in natural environments (Epstein, 2013; Su *et al*., 2013a,b; Awasthi *et al*., 2017). Remarkably, there are few available reports about the effect of Rpf on cellulase activity. In previous studies, the resuscitation and stimulation functions of proteins in the EOM of *M. luteus* have been verified (Su *et al*., 2013a,b, 2015a,b,c,d). This study suggested that Rpf as an efficient additive could significantly promote the growth of cellulolytic bacteria, which will provide a new insight into the potential cellulose‐degrading bacteria within the uncultured microorganisms.

## Conclusions

This paper reported a new method for cellulose degradation using Rpf. The results showed that Rpf could significantly improve cellulase‐producing capability of bacterial community and more culturable cellulose‐degrading bacterial strains. Ten strains which belonged to genera *Bordetella*,* Pandoraea*,* Novosphingobium*,* Bacillus*,* Arthrobacter*,* Nocardiopsis* and *Mycobacterium* were isolated after Rpf addition. In particular, the stimulation and resuscitation functions of Rpf on uncultured *Actinobacteria* with high FPCase activity were tentatively established. Based on the above results, utilization of Rpf in resuscitating these uncultured microorganisms provides new insight into the underlying cellulolytic consortium which lies in improvement of cellulose degradation.

## Experimental procedures

### Production of recombinant Rpf protein

*Micrococcus luteus* IAM 14879 (= NCIMB 13267) used in this study had previously been described (Mukamolova *et al*., 1998; Su *et al*., 2015a,b,c,d). The pure culture was inoculated in modified lactate minimal medium (LMM) of Kaprelyants and Kell (1992). *Micrococcus luteus* genomic DNA was extracted using the EZ‐10 spin column genomic DNA miniprep kit (Bio Basic, Canada) according to the manufacturer's instructions and then was amplified by PCR using primers *rpf*‐F 5′‐ GCGC**GGATCC**ATGGACACCATGACTCTCTTCACC‐3′ and *rpf*‐R 5′‐GATC**AAGCTT**TCAGGCCTGCGGCAGGACGAGCTCC‐3′ (restriction sites included for cloning purpose are in bold italics) and the following conditions: 94°C for 3 min (initial denaturation), 30 cycles of denaturation at 94°C for 30 s, annealing at 57°C for 30 s and 72°C for 1 min and finally 72°C for 8 min. The PCR products were purified and ligated into the NedI/BamHI‐treated pET‐28a vector and then transformed into chemically competent *E*. *coli* BL21 (DE3) cells. To make protein containing an N‐terminal histidine tag, the recombinant cells were cultured with kanamycin (50 μg/ml) and induced by isopropyl‐β‐d‐thiogalactopyranoside (IPTG, 100 μg/ml) as previously described (Mukamolova *et al*., 1998). Cells were harvested by centrifugation (7104 g, 15 min) and washed twice in phosphate‐buffered saline (PBS). After sonication in Tris‐HCl (25 mM), the supernatant was obtained by centrifugation, and then applied to Ni‐NTA‐agarose column (Qiagen, Hilden, Germany), equilibrated with binding buffer. The column was washed with 20 volume binding buffer (0.5 M NaCl; 20 mM Tris‐HCl, pH 7.9) followed by 20 volume binding buffer containing imidazole of different concentrations (20–200 mM, increased in steps of 20 mM). The eluate was dialysed against 25 mM Tris‐HCl at 4°C for 17–24 h. The concentration of Rpf protein was determined using Bradford quantification kit (Sangon, Shanghai, China). The molecular mass of the recombinant Rpf protein was assessed by SDS‐PAGE using standard markers (MBI Fermentas, Vilnius, Lithuania; Mukamolova *et al*., 1998, 2002). Finally, the purified protein was stored at −20°C within 1 week for further experiments. The peptidoglycan hydrolase activity of Rpf was investigated by a turbidimetric assay with *M*. *luteus* cell wall as substrate. Peptidoglycan solubilization was followed by monitoring the turbidity decrease in the reaction mixture at 600 nm (OD_600_) with a spectrophotometer (Santin and Cascales, 2017). A similar assay was performed in a control group without Rpf protein addition. Turbidimetric assay of peptidoglycan solubilization by Rpf protein was performed in triplicate experiments.

### Sample collection and bacterial community analysis

Mature compost samples produced from agro‐industrial and household wastes were collected from a composting centre in Jinhua, China. Triplicate biological samples (HWC1, HWC2 and HWC3) were taken from composting mixture at different locations. Microbial DNA was extracted from compost samples using the EZNA Soil DNA Kit (Omega Bio‐Tek, Norcross, GA, USA). The V3–V4 region of the bacterial 16S rRNA gene was amplified by PCR (95°C for 2 min, followed by 25 cycles at 95°C for 30 s, 55°C for 30 s, and 72°C for 30 s and a final extension at 72°C for 5 min) using primers **341F** (5′‐barcode‐ CCTAYGGGRBGCASCAG)‐3′ and **806R** (5′‐GGACTACNNGGGTATCTAAT‐3′) (Yu *et al*., 2005), where the barcode was an eight‐base sequence unique to each sample. The 20 μl PCR mixture contained 4 μl of 5× FastPfu buffer, 2 μl of 2.5 mM dNTPs, 0.8 μl of each primer (5 μM), 0.4 μl of FastPfu Polymerase and 10 ng of template DNA. Amplicons were extracted from 2% agarose gels and purified using the AxyPrep DNA Gel Extraction Kit (Axygen Biosciences, Union City, CA, USA) and quantified using QuantiFluor™ (Promega, Madison, WI, USA). Purified PCR products were quantified by Qubit 3.0 Fluorometer (Life Technologies, Gaithersburg, MD, USA), and every 24 amplicons whose barcodes were different were mixed equally. All the amplicons were used to construct Illumina pair‐end libraries. Sequencing was conducted on an Illumina HiSeq 2500 platform. Raw sequencing data were deposited in the NCBI Sequence Read Archive (SRA) database with accession number SRP109740.

The raw sequence data, as FASTQ files, were demultiplexed using the barcode sequence with the exact barcode matching parameter. Low‐quality base pairs were removed using Trimmomatic (version 0.36; Bolger *et al*., 2014). Paired‐reads were merged using USEARCH fastq_mergepairs command (version 9.2.64; Edgar and Flyvbjerg, 2015) with the default parameters. OTUs were clustered with 97% similarity cut‐off using USEARCH UPARSE (Edgar, 2013), and the chimeric sequences were removed in the UPARSE pipeline. The phylogenetic affiliation of each 16S rRNA gene sequence was analysed by USEARCH SINTAX algorithm against the Ribosomal Database Project (RDP) 16S rRNA gene training set (version 16) using confidence threshold of 0.8. The OTUs identified as mitochondrial or chloroplast rRNA sequences were discarded.

### Enrichment and cultivation

The compost sample (3%, w/v) was incubated in enrichment medium containing (per litre): 10 g carboxymethyl‐cellulose (CMC), 1 g K_2_HPO_4_, 2 g peptone, 0.5 g NaCl and 0.5 g MgSO_4_·7H_2_O. The pH was adjusted to 7.0 prior to inoculation. Culture enrichment was cultivated in conical flasks at 30°C on a rotary shaker at 160 rpm. Cultures were transferred to fresh medium every 5 days during 3 weeks (seed volume of 6%, v/v). Throughout the entire enrichment period, an equal amount of Rpf (0.25%, v/v) and inactivated Rpf (sterilized at 121°C for 15 min) were added to the TG and CG respectively. Each experiment was performed in three replicates.

### Bacterial isolates and phylogenetic analysis

The TG and CG were diluted respectively in 10‐fold steps, according 1 ml of the previous dilution to 9 ml of sterilized water. Then 0.2 ml of serial dilutions (from 10^2^‐fold to 10^8^‐fold) was plated on CMC‐Congo red agar for enumeration of cellulose‐degrading bacteria using a modified Hendricks method (Hendricks *et al*., 1995). Morphologically different colonies with a halo zone were selected and purified. Genomic DNA was extracted from the pure bacterial cultures using the EZ‐10 spin column genomic DNA miniprep kit (Bio Basic Inc., Toronto, Canada) according to the manufacturer's instructions. The DNA extracts were amplified by PCR using primers 8F/1541R as described previously (Su *et al*., 2013a,b). Based on combined morphology and 16S rDNA sequence analysis, isolates that were unique in the TG, while no counterparts in the CG, were selected for further analysed. Phylograms were constructed with the MEGA7 program (Kumar *et al*., 2016). The 16S rDNA sequences of the remaining strains which were existed in both TG and CG were submitted to EzbioCloud database for comparison and identification. All sequences reported in this study were submitted to NCBI GenBank under the following accession numbers MF136413–MF136427 and MF442430–MF422435.

### Measurement of enzyme activities

The enrichment cultures from TG and CG, as well as the cultures from selected isolates, were centrifuged (7104 g, 15 min) and washed twice with sterile NaCl (0.85%, w/v). The washed pellets were resuspended in the same volume of sterilized NaCl solution. The cell suspensions (10^8^ cells/ml) were cultured at 37°C at 160 rpm in an enzyme production media containing (per litre) 10 g avicel cellulose, 2 g (NH_4_)_2_SO_4_, 2 g NaCl, 2 g MgSO_4_·7H_2_O and 4 g CaCO_3_. The pH was adjusted to 7.0. After 4 days, the two cultures suspensions were centrifuged, and the supernatants were collected as crude enzyme preparation, respectively, for further enzyme assays. The extracellular cellulase activity of FPCase and CMCase was measured in the enzyme assay mixture containing CMC and What‐man no. 1 filter paper respectively. The mixture was incubated at 60°C for 15 and 30 min, respectively, for FPCase and CMCase assay using 3,5‐dinitrosalicylic acid (DNS) method (Manfredi *et al*., 2015). The enzymatic activities were defined in international unit (IU). One IU of enzymatic activity is defined as the amount of enzyme that releases 1 *μ*mol reducing sugars (measured as glucose) per ml per minute. All experiments were carried out in biological triplicates. Error bars represent standard deviation (SD) of the mean of triplicate samples.

## Conflict of interest

None declared.

## Supporting information


**Table S1.** Analysis of top‐hit species of the remaining 11 strains by retrieving the most closely related strains from EzBioCloud database.
