# Supplementary material for: Resuscitation of viable but non‐culturable bacteria to enhance the cellulose‐degrading capability of bacterial community in composting
Source: Microb Biotechnol. 2018 Mar 14;11(3):527–36. doi: 10.1111/1751-7915.13256 (PMC5902322; doi:10.1111/1751-7915.13256)
Supplement: Supplementary file 1 — Table S1. Analysis of top‐hit species of the remaining 11 strains by retrieving the most closely related strains from EzBioCloud database. [file MBT2-11-527-s001.docx]

**Table S1.** Analysis of top-hit species of the remaining 11 strains by retrieving the most closely related strains from EzBioCloud database

| **The strains** | **Accession numbers** | **Base pairs（bp）** | **The most closely related strains** | **Accession numbers** | **Similarity（%）** |
| --- | --- | --- | --- | --- | --- |
| ZS2N662 | MF136413 | 1387 | *Rhodanobacter caeni* MJ01 | GQ250431 | 98.41 |
| ZS2N663 | MF136414 | 1321 | Sphingobium cloacae NBRC 102517 | BCUM01000103 | 97.78 |
| ZS2N664 | MF136415 | 1350 | Brachybacterium nesterenkovii CIP104813 | FWFG01000034 | 99.33 |
| ZS2N65 | MF136416 | 1385 | *Rhodanobacter lindaniclasticus* RP5557 | AF039167 | 98.33 |
| ZS2N64 | MF136417 | 1336 | *Sphingobium wenxiniae* JZ-1 | FJ686047 | 98.19 |
| ZS2N6 | MF136418 | 1390 | *Bacillus halotolerans* ATCC 25096 | LPVF01000003 | 99.86 |
| ZS2N561 | MF136419 | 1372 | *Luteimonas mephitis* DSM 12574 | AULN01000012 | 98.76 |
| ZS2N562 | MF136420 | 1373 | *Bordetella petrii* DSM 12804 | AM902716 | 100 |
| ZS2N55 | MF136421 | 1371 | *Bacillus atrophaeus* JCM 9070 | [AB021181](https://www.ncbi.nlm.nih.gov/nuccore/AB021181) | 99.27 |
| ZS2N1 | MF442430 | 1397 | *Bacillus zhangzhouensis* DW5-4 | JOTP01000061 | 99.93 |
| ZS2R9 | MF442433 | 1347 | *Pandoraea pulmonicola* DSM 16583 | CP010310 | 97.40 |
